# Transcriptome analysis identified the mechanism of synergy between sethoxydim herbicide and a mycoherbicide on green foxtail

**DOI:** 10.1038/s41598-020-78290-6

**Published:** 2020-12-10

**Authors:** Tao Song, Mingguang Chu, Jianping Zhang, Rui Wen, Jillian Lee, Bruce D. Gossen, Fengqun Yu, Gary Peng

**Affiliations:** 1 Saskatoon Research and Development Centre, Agriculture and Agri-Food Canada, 107 Science Place, Saskatoon, SK S7N 0X2 Canada; 2 Present Address: Syngenta Biotechnology China Co., Ltd., Beijing, 102206 China; 3 Present Address: Anhui Academy of Agricultural Sciences, Hefei, 230041 Anhui China; 4 Present Address: China National Rice Research Institute, Hangzhou, 310006 China

**Keywords:** Molecular biology, Plant sciences

## Abstract

Certain synthetic herbicides can act synergistically with specific bioherbicides. In this study, a sethoxydim herbicide at 0.1× label rate improved biocontrol of herbicide-sensitive green foxtail (*Setaria viridis,* GFT) by *Pyricularia setariae* (a fungal bioherbicide agent), but did not change the efficacy on a herbicide-resistant GFT biotype. Reference transcriptomes were constructed for both GFT biotypes via de novo assembly of RNA-seq data. GFT plants treated with herbicide alone, fungus alone and herbicide + fungus were compared for weed-control efficacy and differences in transcriptomes. On herbicide-sensitive GFT, sethoxydim at the reduced rate induced ABA-activated signaling pathways and a bZIP transcription factor 60 (TF bZIP60), while improved the efficacy of biocontrol. The herbicide treatment did not increase these activities or improve biocontrol efficacy on herbicide-resistant plants. An exogenous application of ABA to herbicide-sensitive plants also enhanced bZIP60 expression and improved biocontrol efficacy, which supported the results of transcriptome analysis that identified the involvement of ABA and bZIP60 in impaired plant defense against *P. setariae*. It is novel to use transcriptome analysis to decipher the molecular basis for synergy between a synthetic herbicide and a bioherbicide agent. A better understanding of the mechanism underlining the synergy may facilitate the development of weed biocontrol.

## Introduction

*Setaria viridis* (L.) Beauv. (green foxtail, GFT) is a serious weed problem worldwide^1^ and one of the most abundant agricultural weeds on the Canadian prairies. Sethoxydim, an ACCase inhibitor that catalyzes initial fatty acid biosynthesis^2^ and disrupts the electron transport of photosystem II in the plants of Poaceae^3^, is a synthetic herbicide that has been widely used for management of GFT in Canada. Its efficacy, however, is challenged by the emergence of herbicide-resistant GFT biotypes^4–6^.

In a previous study, a host-specific fungal pathogen, *Pyricularia setariae* Nisikado, was efficacious against GFT seedlings, including the resistant biotype^7^. Applying sethoxydim at sub-lethal doses prior to or with *P. setariae* substantially improved its efficacy against GFT, extending the efficacy to older GFT plants and additional weedy foxtail species^8^. However, the key connections between the physiological and pathological events in this synergistic interaction were not understood. Other herbicides have also been reported to synergize weed biocontrol by fungal pathogens (mycoherbicides). For example, co-application of *Colletotrichum coccodes* with thidiazuron herbicide to velvetleaf (*Abutilon theophrasti* Medic.) increased fungal infection and weed suppression compared to either component alone^9^. Similarly, application of 2,4-D + MCPP at sublethal rates synergized biocontrol of field bindweed (*Convolvulus arvensis* L.) by *Phoma proboscis* Ormeno^10^, and a sublethal dose of glyphosate increased infection success by *Alternaria cassiae* Jurair & Khan in sicklepod (*Cassia obtusifolia* L.)^11^. It has been suspected that these herbicides suppress plant defense responses, facilitating infection and weed-control efficacy by mycoherbicide agents^12^, but the underlying mechanism(s) of these synergistic interactions were not known. Understanding the physiological and molecular basis of this chemical-microbial interaction may stimulate the development of novel approaches and strategies for weed biocontrol.

Genome sequence information makes it possible to study host–pathogen interactions at molecular level^13–15^. Transcriptomes constructed via RNA sequencing (RNA-seq) have been reported for several plant species^16–19^, but the information is generally lacking for most of weed species. This might have limited the critical understanding of molecular mechanisms for weed biocontrol. The genome sequence of foxtail millet (*Setaria italica*) provides genomic insights into grasses, which may be used as a reference in studying GFT because both species belong to the same genus. In this study, reference transcriptomes for herbicide-sensitive (HS) and -resistant (HR) GFT biotypes were constructed using de novo assembly of RNA-seq sequence data. The main objective was to explore the underlying mechanism of the synergy between sethoxydim at a sublethal dose and the mycoherbicide agent *P. setariae* in biocontrol of GFT using transcriptome analysis based on the reference transcriptomes for GFT developed.

## Methods

### Preparation of plants and fungal inoculum

Two GFT biotypes, one sensitive (HS) to ACCase-inhibitor herbicides and the other resistant (HR), were assessed in this study. Both biotypes were collected from crop fields in the province of Manitoba, Canada and identified to species by Dr. Hugh Beckie (formerly AAFC-Saskatoon Research and Development Centre). No permits were required for this collection. The HR trait was confirmed by treating plants at the 3- to 5-leaf stage with the herbicide sethoxydim (Post Ultra) at 1×, 2× and 3× label-recommended rates in a greenhouse. Also, seed increase of both biotypes was carried out in the greenhouse. The resistance mechanism for the HR population has not been determined, but altered ACCase-I isoform and/or impaired electron transport in photosystem II have been reported with other GFT populations resistant to ACCase inhibitors^12,20^. The reason to select the ACCase-inhibitor sensitive and resistant GFT for this study was the availability of both biotypes and synergistic interaction of the herbicide with *P. setariae* in GFT biocontrol in a preliminary study.

For de novo assembly analysis, seed of both biotypes was planted in Sunshine #3 potting mix (SunGro Horticulture, Vancouver, BC) amended with 1% (w/v) of 16–8–12 (N:P:K) control-released fertilizer in 15 cm-diam. pots, and kept in a greenhouse (17–20 °C) with a 14-h period of supplementary lighting at 230 µE/m2s at the canopy level. In addition, seeds were germinated on wet filter paper in Petri dishes for leaf samples of 7-day-old seedlings. For exogenous ABA treatment, HS seeds were germinated on filter paper and transferred at 3 days after germination to growth medium in pots (described above) at 25 plants per pot.

An isolate of *P. setariae* 94-409A, highly virulent to both HS- and HR-GFT biotypes^21^, was used throughout the study. Conidia (spores) were produced on oatmeal agar at 26 °C with a 14-h photoperiod under near-ultra violet light. Spores were collected and adjusted to 1 × 10^6^ spores/ml as described previously^8^. Briefly, the colony was flooded with sterile water, spores dislodged by scraping the surface of the colony, the spore suspension collected, and spore concentration adjusted based on estimates made using a haemocytometer. The spore suspension (inoculum) was stored at 4 °C until use and amended with 0.1% (v/v) Tween 80 (Fisher Scientific, Fair Lawn, NJ) prior to plant inoculation.

### Synergy experiment

To confirm the synergy between sethoxydim and *P. setariae* for biocontrol of GFT, four treatments were applied to each GFT biotype: control (mock: water), herbicide only, *P. setariae* only, and herbicide + *P. setariae* (synergy). A randomized complete block design was used, with four replicates (pots), and each pot of plants was an experimental unit.

At the 3-leaf stage (the 3rd leaf was fully expanded while the 4th leaf was emerging), pots of GFT seedlings were sprayed with 0.1 × sethoxydim (0.3 ml Poast Ultra in 1L water, amended with the adjuvant Merge at 0.01%), at 10 ml/m^2^ from a distance of ~ 50 cm. This application was equivalent to 1/10 of the label-recommended rate of sethoxydim at a carrier volume of 100 L/ha. This treatment was selected to cause only slight damage to sensitive green foxtail^6^. Herbicide-treated plants were placed in the greenhouse for 24 h prior to inoculation with *P. setariae*. The conidial suspension was applied at about 3 ml per pot to result in near runoff coverage on the foliage. Both herbicide and *P. setariae* were applied using an airbrush sprayer (Pasche Airbrush, Harwood Heights, IL) at 275 k Pa constant air pressure. Inoculated plants were placed in a dew chamber (Percival Scientific, Perry, IA) at 20 ± 2 °C in darkness for 24 h for fungal infection to occur, and then moved to the greenhouse for symptoms to develop. At 2 days post-inoculation (dpi), the top three leaves from control and treated plants were sampled for RNA extraction (detailed below).

Treated plants were assessed between 4 and 7 dpi for weed-control efficacy based on infection severity and/or fresh weight. A previous study showed that plant fresh and dry weight provided the same results for weed control assessment^8^, so only fresh weight was assessed in the current study. Fresh weight was assessed at 7 dpi (generally the time of maximum weed reduction) by cutting all plants in a pot at the soil level. Both healthy and dead tissues were weighed, and the mean plant weight calculated.

### Development and assembly of the reference genome

To develop a reference transcriptome for GFT, leaf samples from multiple growth stages were used for each biotype. Plants were established and maintained in a greenhouse as described previously, and the top leaves were sampled at 7, 24 (3-leaf), 42 and 67 (early maturity) days after emergence. Samples from 5 plants of each pot were bulked as a biological replicate, and three biological replicates were produced for each of the growth stages of each biotype.

RNA was extracted form the leaf samples using an RNeasy Plant Mini Kit (Qiagen, Toronto, ON) with on-column deoxyribonuclease (DNase) digestion using a RNase-Free DNase set (Qiagen) following manufacturer’s protocols. The RNA concentration and quality were checked using a Nanodrop 2000c (Thermo Scientific, Wilmington, DE) and a Bio Rad Experion automated electrophoresis system (Bio Rad, Mississauga, ON), respectively. The RNA quality index (RQI) from the Experion assay was used to determine the integrity of RNA samples, and each sample had a RQI > 9.

For each replicate, an equal amount of RNA was taken from the bulked sample of leaves collected at each growth stage (separately for HS and HR), mixed, and used as a template to construct RNA-seq libraries. To determine the best combination of parameters, a total of 30 assemblies were constructed first using HS clean reads to determine the choice of word size (K-mer), length and similarity fractions. Assemblies with K-mer at 64, length and similarity fractions at 0.95 were selected for subsequent analysis of both HS and HR. RNA-seq assays for de novo assembly and transcriptome characterization were performed on an Illumina Miseq benchtop sequencing system using the reagent kit V3-150 cycles (Illumina Inc. San Diego, CA). The sequencing libraries were prepared using TruSeq RNA Sample Preparation Kits v2 (Illumina).

The raw reads were analyzed and comparative BLAST searches were run using CLC Genomics Workbench (CLC-GW) version V8.5 (Qiagen). The raw reads were filtered to remove low-quality reads (> 5% unknown bases or > 50% of the bases with a quality < 5), to generate a set of clean reads. In total, 30 de novo assemblies of GFT transcriptome were constructed using three major parameters (K-mer, length fraction and similarity fraction, Supplementary Table S1) as variables. Use of CLC-GW as an assembler lowered the chance for chimeras^22^.

The protein sequences of *S. italic* (L.) Beauv (foxtail millet) from Phytozome V10.1 (www.phytozome.net) were imported into CLC-GW as the database for BLAST searches. The BlastX searches of assembled contigs were performed with the e-value at 0.01 for the major parameters. The assembly parameter combination that optimized the quality of assembly (K-mer at 64, length and similarity fractions at 0.95) was used to construct the de novo assembly of transcriptome.

In addition to *S. italica*, protein sequences from three other crop species of Poaceae (*Panicum virgatum* L., *Zea mays* L. and *Sorghum bicolor* (L.) Moench ssp. *bicolor*) were also retrieved from Phytozome V10.1, and imported into CLC-GW. They were combined with those of *S. italica* as a database for BlastX comparisons using assembled contigs as input sequences to identify top hits (e ≤ 0.05, similarity ≥ 70%). Contigs without significant hits were compared against the non-redundant (nr) protein database at NCBI (http://www.ncbi.nlm.nih.gov). Pairwise tBlastn comparisons were performed to assess the conservation between GFT and the selected crop species. The same thresholds used in the BlastX searches were applied in tBlastn searches to identify homologous genes.

### Mechanism of synergy

To characterize the transcriptomes of GFT for each treatment in the synergy study, leaf samples were taken at 2 dpi (3 days after sethoxydim treatment) and RNA was extracted and prepared for transcriptome analysis as described previously. RNA-seq clean reads were mapped to the de novo assembled contigs. The transcriptional values were determined for each contig based on RPKM (reads per kilobase of transcriptome per million mapped reads). Gene transcription was characterized by comparing the herbicide, *P. setariae* and synergy (herbicide + *P. setariae)* treatments individually against the control for each GFT biotype (six pair-wise comparisons).

Six groups of differentially expressed genes (DEGs) were examined, with each being separated into up- and down-regulated DEGs. The Empirical Analysis of DEG tool^23^ identified DEGs at a threshold where the absolute value of log2 fold change was ≥ 2^24^. The false discovery rate (FDR) was used to calculate the threshold *P*-value, with a FDR corrected value at *P* ≤ 0.01. Principal component analysis (PCA) was used for exploratory data analysis to make sure the chosen principal factors (treatments) captured a significant amount of variability.

Gene ontology (GO) of the assembled contigs from the HS and HR biotypes was annotated using Blast2GO Pro^15^ to run BlastX algorithms against the NCBI nr database with a taxonomic filter of Viridiplantae (taxid: 33,090). All Blast hits were mapped to functions in the GO database. A GO-term pool generated by GO mapping was used to annotate each of the sequences identified. The statistics of Blast2GO were presented in Supplementary Tables S2 and S3, with the top 20 Biological Process GO terms summarized in Supplementary Fig. S4. To assess the statistical difference in the annotation between UP and DOWN DEGs, an enrichment analysis was performed using the UP-DEGs as a test set and DOWN-DEGs as a reference set based on the Gossip package^25^. A FDR corrected value at *P* ≤ 0.01 was used to filter the GO terms that showed significant differences between the two sets of DEGs. The initial Gossip Results Table was further trimmed to the most specific GO terms.

Reverse-transcription quantitative PCR (RT-qPCR) was used to verify the reliability of RNA-seq data. In that analysis, the log2 fold of RPKM values for 10 highly activated or suppressed defense- or photosynthesis-related DEGs in RNA-seq were compared with the expression ratio (also on log2 scale) of the same set of genes in RT-qPCR 9^15^. The analysis was performed using a StepOne Plus system (Life Technologies, Burlington, ON). RNA samples were prepared as described above. The primers (Supplementary Table S7) were designed using the Applied Biosystems Primer Express V3.0 (Life Technologies) and synthesized by Integrated DNA Technologies Inc. (Coralville, IA). Complementary DNA was synthesized using the Invitrogen SuperScirpt III First-strand Synthesis system (Life Technologies) from 1 µg of total RNA. PCR was conducted using Power SYBR green master mix (Life Technologies) following the manufacturer’s protocols. Cycling conditions were 95 °C for the initial 10 min, followed by 40 cycles of 15 s at 95 °C, 30 s at 50 °C and finally 30 s at 60 °C.

Melt-curve profiling and agarose gel electrophoresis assessments were conducted to ensure the specificity of reaction and absence of primer dimers^15^. The actin gene was used as an endogenous control to normalize the expression level of target genes because of its consistent level of expression among samples tested (data not shown). The relative expression data were analyzed using the StepOne software V2.2.2 (Life Technologies). There were three biological replicates for each treatment and three technical replicates for each cDNA sample tested. The Log2-fold change observed with RT-qPCR was compared with the RNA-seq data. Analysis of variance and Fisher’s Least Significant Difference (*P* < 0.05) were performed using the software Statistical Product and Service Solutions (V20.0; IBM Canada, Markham, ON) to compare the gene transcription level in RT-qPCR.

### Effect of ABA

The effect of ABA on the efficacy of *P. setariae* was investigated using the same experimental design and assessment protocol described previously for the synergy study, except that the ABA was added. The ABA treatment was applied via soil drench around individual GFT plants at 500 µM per plant at the 3-leaf stage, 24 h prior to *P. setariae* inoculation. The ABA stock solution (50 mM) was prepared by dissolving analytical grade ABA (Sigma, Oakville, CA) in 1-N NaOH before being diluted to the target concentration with water. The final concentration of NaOH was about 0.01 N, so a NaOH solution at 0.01 N (no ABA) was applied as a soil drench as a control against the ABA treatment. Leaf samples were collected at 2 dpi for subsequent RT-qPCR analysis for the transcriptional level of bZIP60 (Contig_GFT-HS_9248), substantially upregulated with ABA-activated signaling pathways by sethoxydim in the RNA-seq analysis.

### Data analysis

All efficacy experiments were repeated. Plant fresh-weight data was analyzed using the software Statistical Analysis System v. 9.3 (SAS Institute, Cary, NC, USA). The normality of data was examined with the Shapiro–Wilk Test and the data from repeated trials tested with the Bartlett’s Test before pooling them for the analysis of variance (ANOVA). All data comforted the requirements. Treatment means were separated with LSD (*P* < 0.05) when ANOVA showed significance also at the *P* < 0.05 level.

## Results

### Synergy experiment

**Figure 1:**
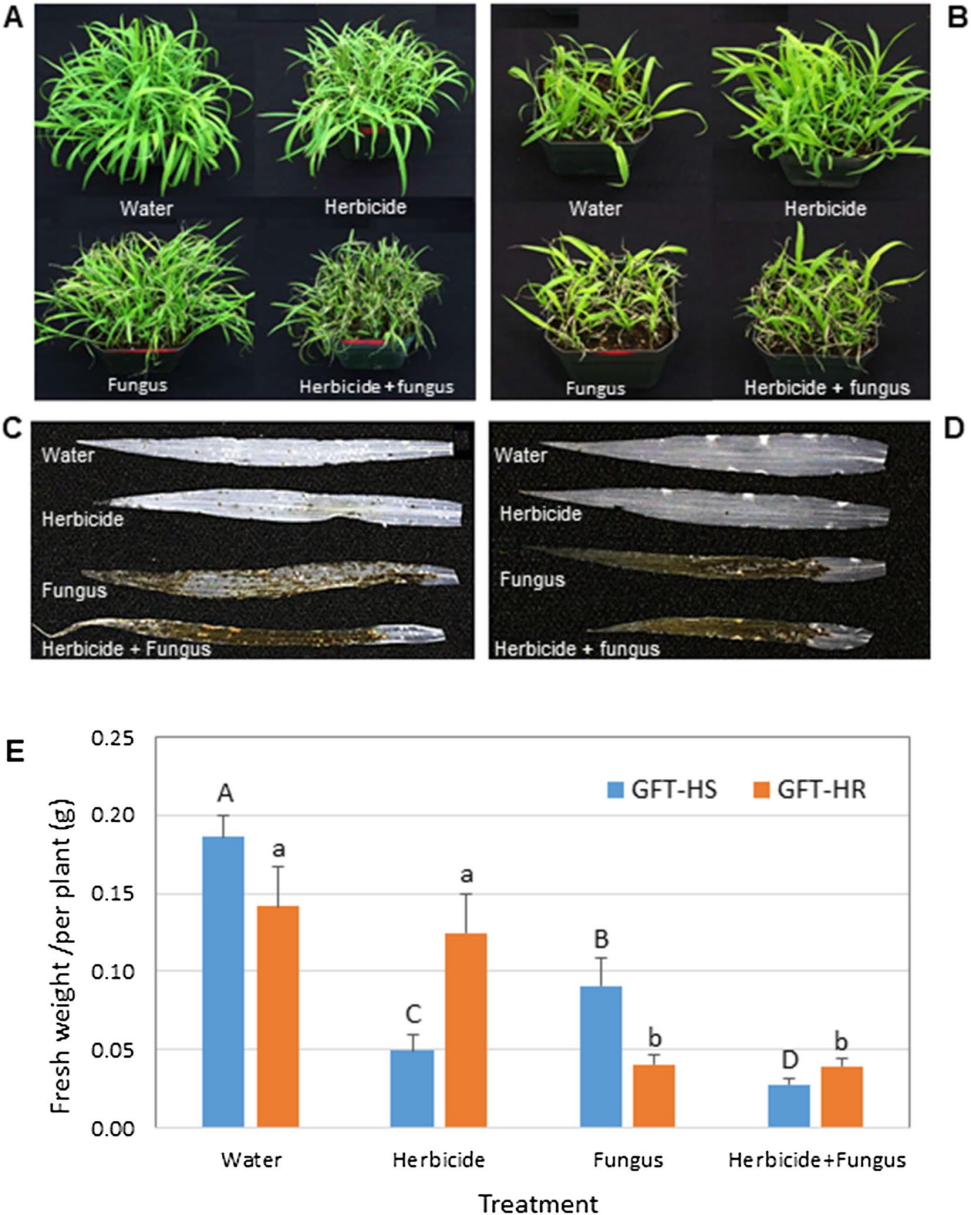
The herbicide sethoxydim at the 0.1 × recommended rate synergizes biocontrol of green foxtail by *Pyricularia setariae* (fungus). (**A**) The effect of herbicide, fungus or herbicide + fungus (synergy) treatment on herbicide-sensitive (HS) green foxtail (GFT) at 4 days post inoculation (dpi). (**B**) Effect of the same treatments on a herbicide-resistant (HR) GFT biotype at 4 dpi. (**C**, **D**) Infection severity on cleared leaves of GFT-HS and GFT-HR biotypes, respectively. (**E**) Fresh weight of variously treated green foxtail plants at 7 dpi. Means with the same capital (HS) or small (HR) letter did not differ (LSD, *P* < 0.05).

When applied at the 0.1 × recommended rate, sethoxydim herbicide was only slightly damaging to HS GFT biotype, with a slightly reduced growth relative to that of control at 4 dpi (Fig. 1A). Inoculation with *P. setariae* resulted in noticeable necrosis on HS GFT plants, while applying the fungus to herbicide-treated plants caused substantially more severe injury than the herbicide alone or fungus alone (Fig. 1A). However, the herbicide caused almost no visible injury to the HR biotype and adding *P. setariae* did not increase the injury over the fungus alone (Fig. 1B). The fungus was more aggressive on HR than on HS plants, with slightly increased infection frequency (Fig. 1C,D). The treatment effects were consistent over the following 3 days on both HS and HR biotypes (Supplementary Figs. S1, S2), with herbicide + *P. setariae* reducing plant fresh weight more substantially on HS GFT than the herbicide alone or fungus alone at 7 dpi (Fig. 1E).

### Development of reference transcriptome

De novo assembly of GFT transcriptomes for the HS and HR biotypes were performed to allow transcriptomic analysis of different treatments. Sequencing of the RNA samples prepared at several growth stages of GFT generated 71.91 M and 68.93 M raw reads, respectively, for HS and HR biotypes (Table 1). After removal of low-quality reads, a total of 71.88 M and 68.91 M clean reads were retained for de novo assembly.

**Table 1:** Statistics for the RNA sequencing and de novo assembly of herbicide-susceptible (HS) and -resistant (HR) green foxtail (GFT) biotypes.

Read characteristics	GFT-HS	GFT-HR
Total raw reads^a^	71.91	68.93
Total clean reads^a^	71.88	68.91
Total assembled contigs	29,515	29,137
Average length of contig (bp)	801	762
Maximum length of contig (bp)	12,221	12,873
Minimum length of contig (bp)	167	157
N75 (bp)	631	581
N50 (bp)	1263	1182
N25 (bp)	2021	1904
Total assembled length (bp)	23,651,319	22,211,385
Total mapped reads^a^	64.03 (89.1%)	61.19 (88.8%)
Total reads mapped in pair	56.88	54.06
Total reads mapped in broken pairs	7.15	7.12

^a^Reads =  × 1,000,000.

When K-mer (word size) was specified, varying length or similarity fraction had only small effects on assembly statistics (Supplementary Table S1), based on the analysis of 30 assemblies constructed for the HS biotype. Thus, the length and similarity fractions were set at 0.95 to determine the proper word size. When K-mer was smaller than 64, several long contigs ≥ 15,000 bp were assembled and BLAST searches of these contigs resulted in hits to multiple genes, indicating that these genes might have been mis-assembled. Assemblies using smaller K-mer, however, resulted in substantially increased number of contigs, which would have exceeded the estimated transcriptome size of fully sequenced foxtail millet^26^, a species closely related to GFT. The assemblies with K-mer at 64 had the highest percentage of contigs with at least one hit to a *S. italica* gene. The K-mer analysis was also performed on the HR biotype, and the results were the same as in the analysis of the HS biotype (data not shown). Therefore, the assemblies with K-mer at 64, length and similarity fractions at 0.95 were used for transcriptome sequences with both GFT biotypes.

**Figure 2:**
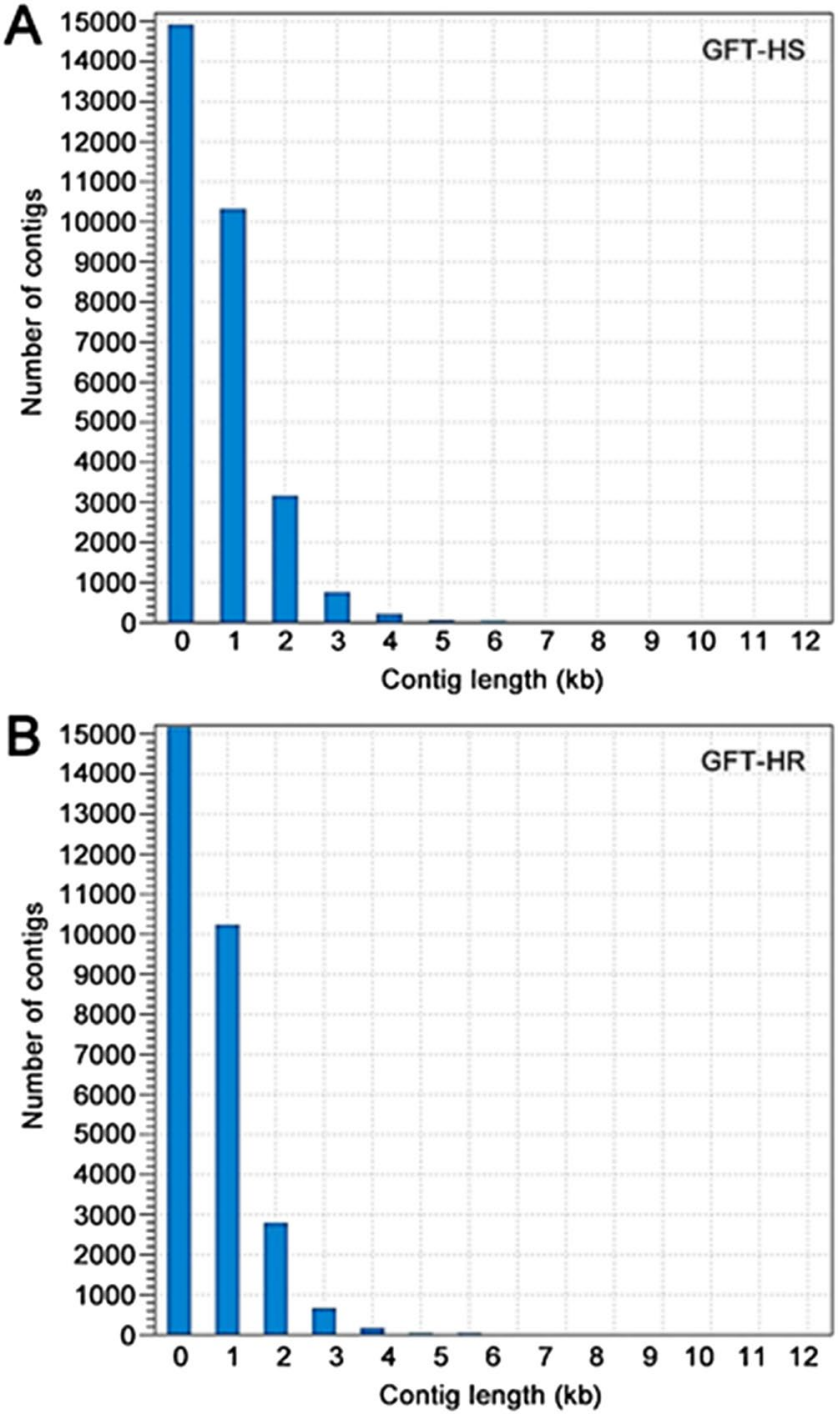
Length and taxonomy distribution of de novo assembled contigs for GFT-HS (**A**) and GFT-HR (**B**).

Using the parameters described above, a total of 29,515 and 29,137 contigs were identified, respectively, for HS and HR GFT (Table 1). The total size of the assembled contigs was 23.65 M bp for the HS biotype and 22.21 M bp for HR, with 89.1% reads mapped to the HS GFT transcriptome, and 88.8% mapped to the GFT-HR transcriptome. The contig N50 was 1263 bp for HS and 1182 bp for HR, respectively (Table 1). Both assemblies showed comparable assembly statistics and size distributions of contigs (Fig. 2A B). For the contigs assembled, 84.6% from HS and 85.5% from HR had at least one hit to the NCBI nr database (Supplementary Tables S2, S3). These results collectively showed a high quality of the transcriptome assemblies.

**Figure 3:**
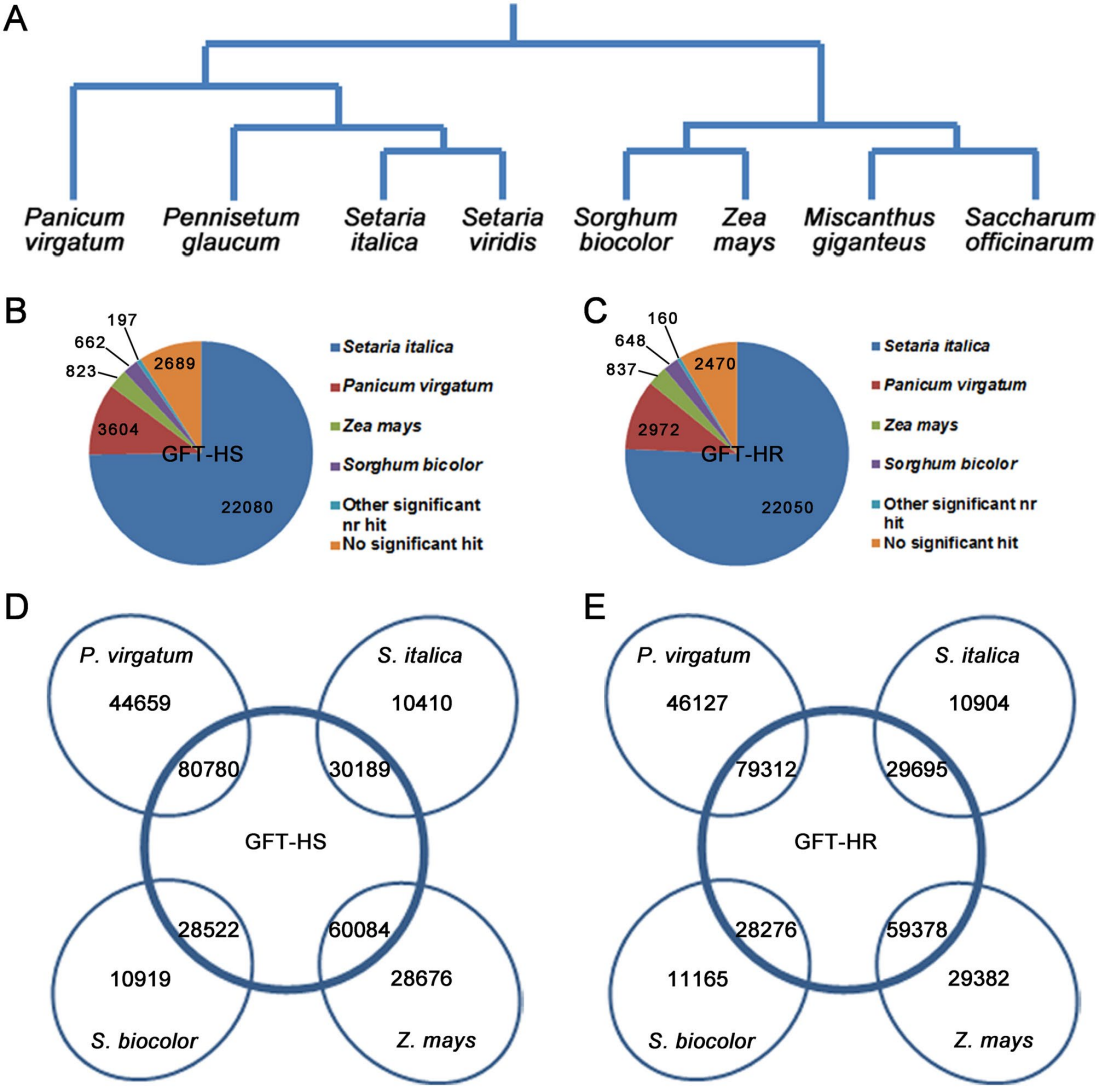
Comparative analysis of de novo assembled transcriptomes of green foxtail with related species in Poaceae. Phylogenic tree for selected species (**A**, adopted from Li and Brutnell^8^), assembled HS GFT (**B**) and HR GFT (**C**) contigs with BlastX top hits to those of *Setaria italica,* *Panicum virgatum,* *Zea mays* and *Sorghum bicolor* (https://phytozome.jgi.doe.gov/pz/-portal.html#). GFT contigs without any hit were compared against the NCBI nr database. tBlastn comparisons of transcriptomes between assembled GFT-HS (**D**) or GFT-HR (**E**) with those of selected species. The value in each of the overlapping areas represents the number of genes with significant homology (e ≤ 0.05, match ≥ 70%) in the GFT transcriptomes.

To assess the completeness of transcriptome sequencing, the assembled GFT transcriptomes were compared with those of fully sequenced species in Poaceae (Fig. 3A), including *Panicum virgatum*, *Setaria italica*, *Sorghum bicolor* and *Zea mays*. The BLAST search had 22,080 hits (e ≤ 0.05) to genes in *S. italica* for HS and 22,050 hits for HR transcripts (Fig. 3B,C). There were only ~ 3000 hits to *P. virgatum*, ~ 800 and ~ 600 hits to the transcripts of the other two species, respectively (Fig. 3B,C). The hit numbers are clearly related to the phylogenetic distance between GFT and the other grass species, with *S. italic* being closest to GFT and *S. bicolor* and *Z. mays* being much further apart^27^. Pairwise tBlastn comparisons showed a similar pattern; there was a much higher proportion of genes in *S. italica* that had homologs in the HS or HR transcriptome than for the other two grass species (Fig. 3D,E). These comparisons showed that 73%, 63%, 71% and 66% of the genes identified in GFT-HS or GFT-HR were homologous to those in *S. italica*, *P. virgatum, Sorghum bicolor* and *Z. mays*, respectively (Supplementary Table S4).

The GFT transcripts were annotated using Blast2GO PRO^28^ against the NCBI nr database (Supplementary Tables S2 and S3). In the taxonomic distributions of the HS and HR transcriptomes (Supplementary Fig. S3A, S3B), more than 75% of the transcripts had top hits to the genes in *S. italica* but only 7% to genes in *S. bicolor* and *Z. mays*. Outside Poaceae, fewer than 600 GFT transcripts had a hit and ~ 15% of the transcripts either had hits to non-plant sequences or lacked similarity to any of the sequences in the database. The statistics on Blast2GO were presented in Supplementary Fig. S3C, S3D, S3E and S3F. The top 20 GO terms on biological processes annotated for HS and HR transcriptomes were shown in Supplementary Fig. S4.

Both foxtail millet and GFT are excellent model C4 plants, with carbonic anhydrase, malate dehydrogenase, NADP-dependent malic enzyme, phosphoenolpyruvate carboxylase, phosphor-enolpyruvate carboxylase kinase and pyruvate orthophosphate dikinase involved in photosynthesis^29^. These genes were identified in the assembled transcriptomes of HS and HR biotypes, with a similar number of copies (Table 2). They were also reported in foxtail millet^14^, except the phosphoenolpyruvate carboxylase kinase. This result, together with those of BLAST searches, indicated a reasonable coverage by the GFT transcriptomes assembled that provides suitable references in subsequent RNA-seq analysis.

**Table 2:** Genes involved in C4 biosynthesis in herbicide-susceptible (HS) and -resistant (HR) biotypes of green foxtail (GFT).

Enzymes	GFT-HS			GFT-HR		
	Copies	Contig #	RPKM^a^	Copies	Contig #	RPKM^a^
Carbonic anhydrase	4	76	2007.33	4	203	2120.25
Malate dehydrogenase	5	86	543.910	5	163	550.76
NADP-dependent malic enzyme	7	584	1491.58	8	77	1492.11
Phosphoenolpyruvate carboxylase	9	169	2234.78	8	48	2225.96
Phosphoenolpyruvate carboxylase kinase	4	17,439	13.20	4	13,654	16.50
Pyruvate rthophosphate dikinase	2	10	2689.54	4	69	2851.67

^a^RPKM = Reads Per Kilobase of transcriptome per million Mapped reads.

### Transcriptome analysis of the synergy experiment

**Figure 4:**
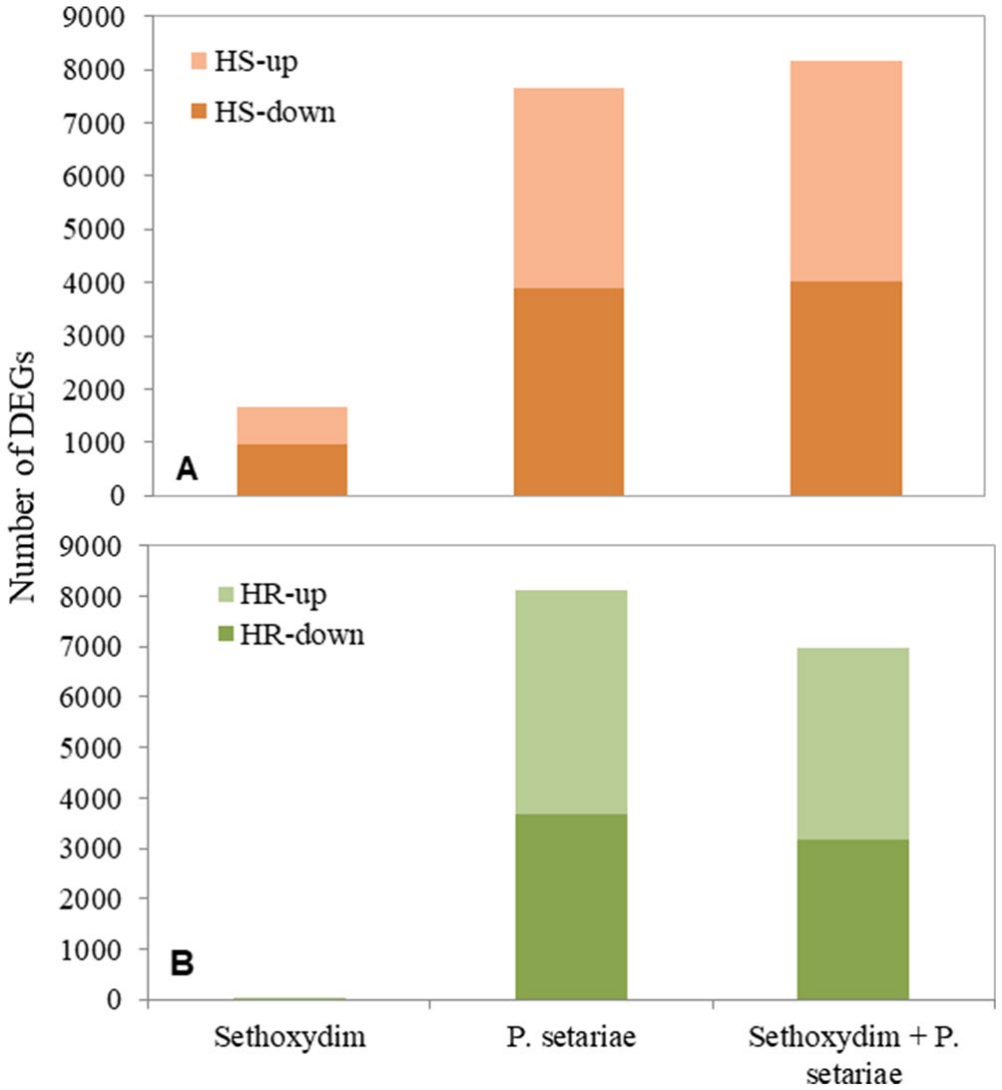
Number of up- and down-regulated differentially expressed genes (DEGs) obtained for HS- (**A**) and HR-GFT (**B**) under the sethoxydim, *P. setariae* and sethoxydim + *P. setariae* treatments. DEGs were based on the comparison against the reference GFT genomes constructed by de novo assembly.

An RNA-seq analysis of samples from HS and HR biotypes in the synergy study included these treatments: control, sethoxydim (herbicide) only, *P. setariae* only and herbicide + *P. setariae* (synergy) for each biotype. For HS GFT samples, 23.66 M, 24.31 M, 25.39 M and 23. 80 M reads were obtained from the respective treatments; for HR-GFT, 25.90 M, 28.75 M, 28.56 M and 27.25 M reads were obtained (Table 3). After filtering, these reads were aligned to the reference transcriptome using CLC-GW. The transcriptomes in these treatments matched approximately 93% of the contigs assembled (Supplementary Tables S5, S6). Differentially expressed genes (DEGs) were identified against the reference transcriptomes (developed earlier in the study) based on RPKM, fold change ≥ 2 and FDR-corrected *P*-value at 0.01 (Supplementary Tables S5, S6). The numbers of up- and down-regulated DEGs were obtained for the four treatments on both GFT biotypes (Fig. 4). On HS, most DEGs were identified with the synergy treatment, while on HR most DEGs were identified with the *P. setariae* alone treatment (Fig. 4).

**Table 3:** Statistics of RNA-seq for herbicide-sensitive (HS) and—resistant (HR) biotypes of green foxtail (GFT) treated with sethoxydim herbicide (0.1 × recommended rate), *Pyricularia setariae* (Ps), and herbicide plus Ps (H + Ps).

Treatment	GFT-HS				GFT-HR			
	Control	Herbicide	Ps	H + Ps	Control	Herbicide	Ps	H + Ps
Total Raw reads^a^	23.66	24.31	25.39	23.80	25.90	28.75	28.56	27.25
Total Clean Reads^a^	23.65	24.30	25.38	23.,79	25.90	28.75	28.55	27.24
Total mapped reads^a^	19.94	19.57	19.38	17.24	21.78	24.29	21.18	20.49
Unique reads mapped^a^	19.94	19.57	19.38	17.23	21.78	24.29	21.18	20.49
Non-specific reads mapped^a^	0.88	0.78	0.99	0.86	1.05	1.40	1.48	1.44
Unmapped reads	3.70	4.73	6.00	6.55	4.12	4.46	7.37	6.76
Gene detected	27,940	28,070	27,976	27,747	27,659	27,846	27,713	27,665
DEGs identified	n/a	1667	7654	8141	n/a	46	8002	6970

^a^Reads =  × 1,000,000.

PCA on identified DEGs showed similar patterns among the replicates of each treatment (Supplementary Fig. S5). The herbicide treatment was different from the control in HS but not in HR plants, which indicated that the transcriptome of HR was not altered by the herbicide sethoxydim, likely due to the herbicide insensitivity of the HR biotype.

RT-qPCR was performed on 20 transcripts selected from the HS and HR DEGs to assess the reliability of RNA-seq data. These transcripts were involved either in C4 photosynthesis (HS Contigs 76, 86, 1175, 584, 169, 10, 17,439; HR Contigs 203, 163, 1152, 77, 48, 69, 13,654) or annotated with the GO term ‘abscisic acid (ABA)-activated signaling pathway’ (HS contigs 21,275, 24,431, 9248; HR contigs14285, 3680, 8003), based on significant down or up regulation in RNA-seq of herbicide treatments on HS relative to nontreated control. The fold-change values of these transcripts in PCR (primer sequences: Supplementary Table S7) were similar to those in the RNA-seq data (Supplementary Fig. S6); both results showed that contigs involved in C4 photosynthesis of HS were down-regulated by the herbicide, *P. setariae*, or herbicide + *P. setariae* (synergy). However, the same genes up-regulated by the herbicide in HR were generally down-regulated by the *P. setariae* or synergy treatment. These results indicate that the mass RNA-seq data likely are trustworthy. The results also showed that the HR GFT biotype tolerant to sethoxydim may be resulted from many genes involved in C4 photosynthesis, which are either insensitive to or can be activated by the herbicide treatment.

Enrichment analysis, based on Fisher’s Exact Test in Blast2GO, identified the biological processes associated with up- and down-regulated DEGs. In HS, a total of 139 GO terms showed significant enrichment, with 63 linking to up-regulated and 76 to down-regulated DEGs among the treatments (Supplementary Table S8). In HR, only 36 GO terms were associated with up-regulated and 76 with down-regulated DEGs (Supplementary Table S8). The up-regulated GO terms with potential involvement in signaling, responses to stimulus and disease resistance for both HS and HR are listed in Table 4. GO terms down-regulated most substantially by the fungicide treatment in HS include those associated with photosynthesis and thylakoid membrane organization (Table 5).

**Table 4:** Up-regulated biological processes in herbicide-susceptible (HS) and -resistant (HR) biotypes of green foxtail (GFT).

GO terms—biological Process	Induced in GFT-HS by	Induced in GFT-HR by
Abscisic acid-activated signaling pathway	Synergy	Pathogen/synergy
Activation of MAPKK activity	Pathogen/synergy	Pathogen/synergy
Defense response by callose deposition	Pathogen/synergy	Synergy
Diterpenoid biosynthetic process	Pathogen/synergy	Pathogen
Gibberellin metabolic process	Pathogen/synergy	Pathogen
Lignin biosynthetic process	Pathogen/synergy	Pathogen
Negative regulation of programmed cell death	Pathogen/synergy	Pathogen/synergy
Organophosphate ester transport	Pathogen	Pathogen
Phytoalexin biosynthetic process	Pathogen/synergy	Pathogen/synergy
Protein glycosylation	Pathogen/synergy	Pathogen/synergy
Protein ubiquitination	Synergy	Pathogen/synergy
Regulation of apoptotic process	Pathogen/synergy	Pathogen/synergy
Respiratory burst involved in defense response	Pathogen/synergy	Pathogen/synergy
Response to heat	Herbicide/pathogen/synergy	Pathogen/synergy
Response to hydrogen peroxide	Pathogen/synergy	Synergy
Response to wounding	Pathogen/synergy	Pathogen/synergy
Stress-activated MAPK cascade	Pathogen/synergy	Pathogen/synergy
Transmembrane receptor protein serine/threonine kinase signaling pathway	Pathogen/synergy	Pathogen/synergy

**Table 5:** Down-regulated biological processes in herbicide susceptible (HS) and resistant (HR) biotypes of green foxtail (GFT).

GO Terms	Suppressed in GFT-HS by	Suppressed in GFT-HR by
Carotenoid biosynthetic process	Pathogen/synergy	pathogen/synergy
Cellular response to oxidative stress	Pathogen	Pathogen
Chlorophyll biosynthetic process	Herbicide/pathogen/synergy	Pathogen/synergy
Chloroplast relocation	Herbicide/pathogen/synergy	Pathogen/synergy
Cysteine biosynthetic process	Herbicide/pathogen/synergy	Pathogen/synergy
Glucosinolate biosynthetic process	Pathogen/synergy	Pathogen/synergy
Isopentenyl diphosphate biosynthetic process, methylerythritol 4-phosphate pathway oxidative phosphorylation	Herbicide/pathogen/synergy pathogen	Pathogen/synergy Pathogen/synergy
Photosynthesis, light harvesting	Herbicide/pathogen/synergy	Pathogen/synergy
Photosynthetic electron transport in photosystem I	Herbicide/pathogen/synergy	Pathogen/synergy
Photosystem II assembly	Herbicide/pathogen/synergy	Pathogen/synergy
Protein targeting to chloroplast	Pathogen/synergy	Pathogen/synergy
Reductive pentose-phosphate cycle	Pathogen/synergy	Pathogen/synergy
Regulation of meristem growth	Pathogen/synergy	Pathogen/synergy
Regulation of photosynthesis	Pathogen/synergy	Pathogen/synergy
Regulation of proton transport	Herbicide/pathogen/synergy	Pathogen/synergy
Response to blue light	Herbicide/synergy	Pathogen/synergy
Response to far red light	Herbicide/pathogen/synergy	Pathogen/synergy
Response to red light	Herbicide/pathogen/synergy	Pathogen/synergy
Response to sucrose	Pathogen/synergy	Pathogen/synergy
Stomatal complex morphogenesis	Herbicide/pathogen/synergy	Pathogen/synergy
Sucrose metabolic process	Pathogen	Pathogen/synergy
Thylakoid membrane organization	Herbicide/pathogen/synergy	Pathogen/synergy

ABA-activated signaling pathways and protein ubiquitination were up-regulated exclusively for the synergy treatment in HS plants (Table 4). The enrichment analysis showed that the genes encoding NCED (9-*cis*-epoxycarotenoid dioxygenase) involved in ABA synthesis^30^, were also up-regulated significantly (Supplementary Table S9). For example, the fold change of HS contig16797 (encodes NCED) was about 150, indicating that ABA synthesis was increased substantially in the synergy treatment. Also, transcription level of the basic leucine zipper (bZIP) transcription factors (TFs), which is enhanced by ABA-activated signalling, showed the up-regulation of three bZIP TF superfamily proteins (HS contigs 11,663, 15,665 and 360) with 7.8-, 8.2- and 3.5-fold changes, respectively. Interestingly the bZIP TF 60 (contig 9248), with a 7.1-fold change, showed an unique transcriptional pattern; it was increased by the herbicide at almost twice the expression level relative to that by the pathogen only treatment (Supplementary Fig. S6, Supplementary Table S10).

The synergy treatment also up-regulated protein ubiquitination in HS plants. The transcription level of the ubiquitin activating enzyme (HS contigs 16,970, 3695), ubiquitin-conjugating enzyme (HS contig 4091) and e3 ubiquitin protein ligase (HS contigs 1140, 25,781 and 4878) all increased by at least twofold (Supplementary Table S11). Many of the u-box domain-containing proteins, with ubiquitin-protein transferase activity and usual function as an ubiquitin-protein ligase^31^, were increased as well (SupplementaryTable S11). Interestingly, ABA-activated signaling pathways and protein ubiquitination also increased in the pathogen only treatment on HR, with or without the involvement of herbicide (Table 4).

### Effect of ABA

**Figure 5:**
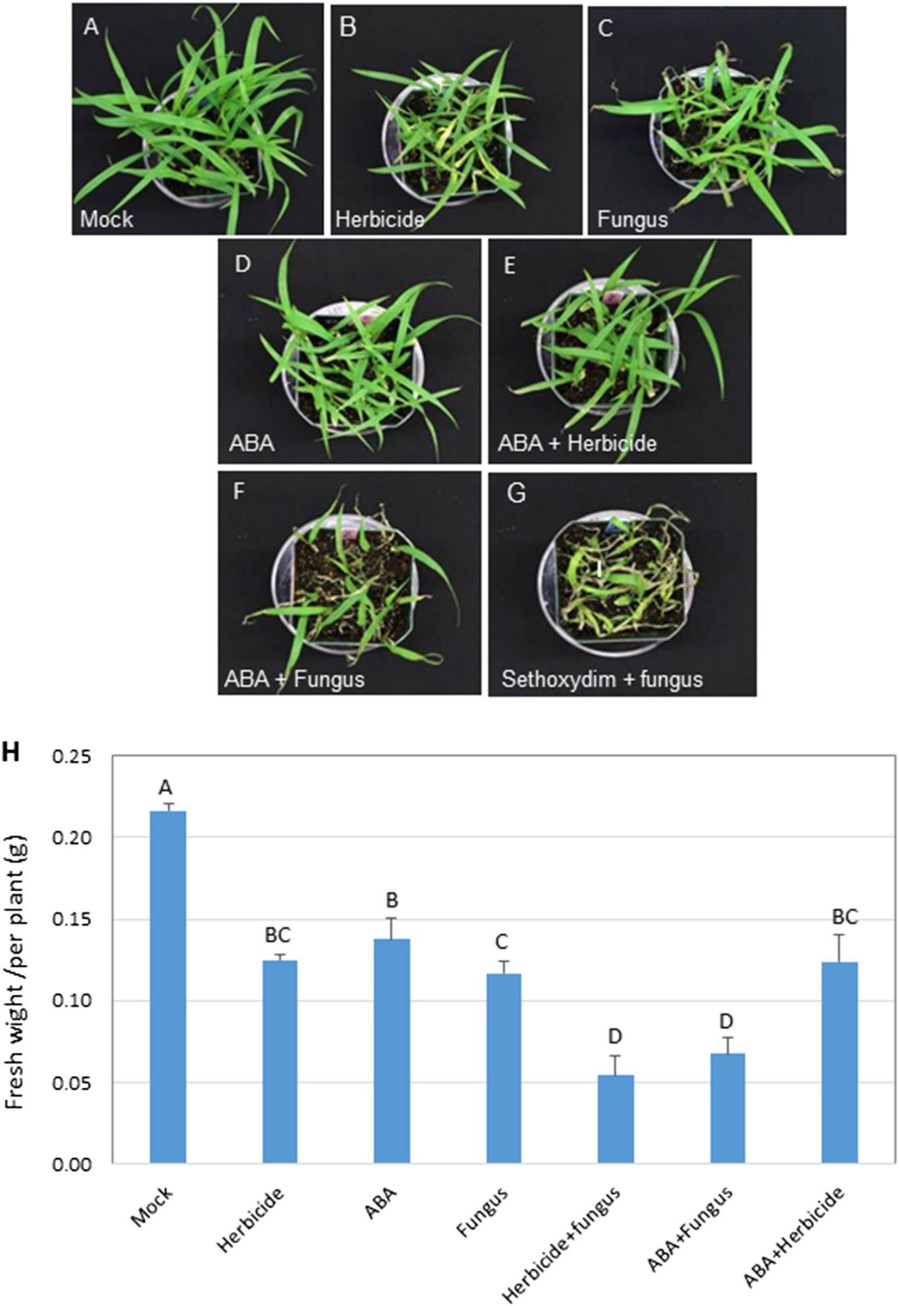

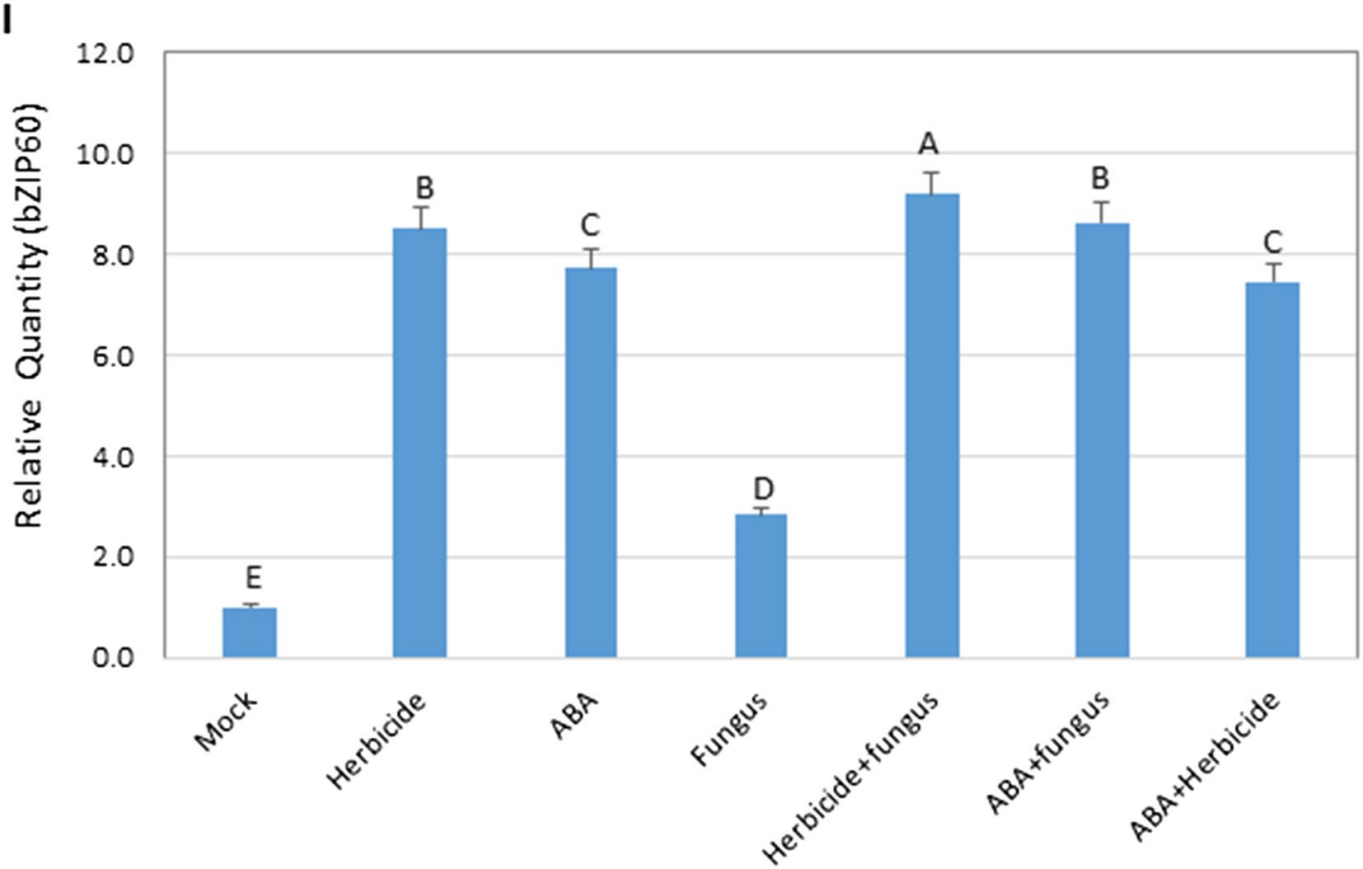
Effect of herbicide sethoxydim and abscisic acid (ABA) on biocontrol of herbicide-sensitive (HS) green foxtail (GFT) with *Pyricularia setariae* (fungus), with symptoms observed at 4 dpi (**A**–**G**) and plant fresh taken at 7 dpi (**I**). The fresh weight was based on an average of 10 plants in each replicate. The transcriptional level of bZIP60 (Contig_GFT-HS_9248) was determined for each treatment with RT-qPCR (H).

To confirm the role of ABA in sethoxydim synergizing the biocontrol of HS GFT by *P. setariae*, an ABA solution was applied as a soil drench prior to inoculation of GFT with *P. setariae*. The ABA treatment reduced the growth of GFT slightly as did by sethoxydim at 4 dpi (Fig. 5A,B, 5D), while no other effect was observed. Combining ABA with sethoxydim did increase the effect relative to either component applied alone (Fig. 5B,D,E). The ABA treatment substantially accelerated the fungal infection on GFT relative to the *P. setariae* inoculation alone (Fig. 5C,D,F), with the disease severity and plant fresh weight similar to those caused by the herbicide + *P. setariae* treatment (Fig. 5F,G,H). The transcription of bZIP60 was also enhanced by ABA to the levels similar to that induced by the herbicide alone (Fig. 5I). Use of ABA with the herbicide did not further increase the transcription of bZIP60 relative to ABA or herbicide alone, which may indicate that both influence the same pathways of bZIP60.

## Discussion

### Transcriptome analysis of synergy for biocontrol

The current study confirmed a previous report^8^ that application of sethoxydim at a sublethal rate improved the efficacy of biocontrol on HS GFT biotype by *P. setariae*. Sadly, this effect was absent on the HR biotype, likely because of the little impact exerted by the herbicide treatment, which is reflected by little change in RNA-seq profile between herbicide-treated and control HR GFT plants. Earlier studies have rarely looked at the molecular mechanisms of weed biocontrol or the synergy of the biocontrol agent with synthetic herbicides, which might be important to the further development of mycoherbicide technologies.

In this study, de novo assemblies of transcriptomes were constructed for both HS and HR GFT biotypes using RNA samples pooled across multiple plant growth stages for maximum coverage^26^. At about 70 M reads and contig lengths at 762–801 bp (Supplementary Table S1), the sequence data is of limited depth, which reflects a limitation of the Miseq sequencing technology used in the study relative to the latest technology. However, the total contig numbers in the de novo transcriptome of GFT are similar to that of the projected transcriptome size based on foxtail millet (*S. italica*) genome sequencing^26^. BlastX and tBlastn analyses of GFT assemblies (HS and HR) against those of fully sequenced Poaceae species also showed a reasonable coverage of homologous sequences (Fig. 3B,C). The quality of de novo transcriptome assemblies should be assessed according to a combination of metrics^22^, including the proportion of reads mapping, recovery of conserved and widely expressed genes, and N50 length statistics. For the contigs assembled, about 85% from HS or HR genotype had at least one hit to the NCBI nr database, indicating that conserved and widely expressed genes were in the assemblies. Based on the consideration of multiple parameters, we believe the assemblies of GFT transcriptomes constructed were of reasonable quality. As expected, the highest proportion of homologous sequences were with *S. italica*, the species most closely related to GFT (Fig. 3B,C).

The transcriptomes for each treatment covered more than 93% of the contigs in the de novo assemblies. Differentially expressed genes were identified in both HS and HR biotypes, based on RPKM values against respective controls. GO terms for biological processes were associated with both up- and down-regulated DEGs in the enrichment analysis (Tables 4 and 5). Together, these data provide some insights into how the herbicide synergizes the biocontrol by *P. setariae* on HS but not HR biotypes.

The herbicide sethoxydim suppresses photosynthesis^32^ in grasses by disrupting the electron transport of photosystem II^12^ and also by blocking fatty acid biosynthesis via ACCase inhibition^33,34^. However, the mechanism underlying the synergy between the herbicide and *P. setariae* in biocontrol of GFT was not known. In the current study, each of the six important genes involved in C4 photosynthesis was identified in the GFT transcriptome assemblies (Table 2). Application of herbicide alone or *P. setariae* alone affected the expression of these genes differentially, depending on the sensitivity of GFT to the herbicide and interaction with *P. setariae*.

All three biological processes involved in photosynthesis, including thylakoid membrane organization, were decreased by the herbicide in the HS biotype but not the HR biotype (Table 5). The thylakoid membrane is where light-dependent reactions occur during photosynthesis and is composed primarily of proteins and lipids, with monogalacto-syldiacylglycerol (MGDG) and diagalactosyldiacylglycerol (DGDG; converted from MGDG) being the key structural lipids^35^. These two galactolipids are associated with both photosystems I and II^36–39^. MGDG-deficient mutants of Arabidopsis exhibited disruption in chlorophyll biosynthesis and photosynthetic activities^40^. The inhibition of ACCase may suppress de novo fatty acid biosynthesis in chloroplast, resulting in a shortage in MGDG, which can causes the collapse of the thylakoid membranes. It is unclear how this suppressed photosynthesis relates to the synergized biocontrol of HS GFT by *P. setariae*.

### Effect of ABA, bZIP60 and protein ubiquitination

The enrichment analysis revealed that ABA-activated signaling and protein ubiquitination pathways were enhanced by the herbicide + *P. setariae* treatment on HS plants but not on HR plants (Table 4). These pathways are of particular interest for biocontrol because of their role in plant response to abiotic^41,42^ and biotic^43^ stresses. In an earlier study, ABA increased the susceptibility of rice plants to blast caused by *P. grisea*^44^, a pathogen closely related to *P. setariae*^45^. It is noteworthy that applying the herbicide or *P. setariae* alone did not enhance these pathways substantially in HS GST; it is possibly the herbicide treatment predisposes HS plants to rapid colonization by *P. setariae*, resulting in much stronger ABA-activated signaling once the infection takes place. In contrast, the herbicide has no direct effect on HR plants (Fig. 1B), so there would be little or no synergy with *P. setariae*.

The transcriptional levels of each DEG associated with the increased ABA-dependent signaling in HS plants were also examined. The contig 9248, which encodes the bZIP60 TF (Supplementary Table S10), was prominently upregulated, with expression levels similar for herbicide and herbicide + *P. setariae* treatments, but twice as high as that for *P. setariae* alone (Supplementary Fig. S6). This upregulation demonstrated that the herbicide treatment enhanced the expression of bZIP60, which may play a role in increasing the efficacy of the biocontrol by acting as a negative regulator of defense response to infection by *P. setariae*. The contig 3680 encodes a bZIP60 gene in the HR biotype, but its transcription was not affected by the herbicide treatment. However, application of *P. setariae* alone (Supplementary Fig. S6) increased transcription levels of bZIP60 gene in the HR biotype similar to those in herbicide-treated HS biotype. This may explain why *P. setariae* alone was more aggressive on HR than on HS plants (Fig. 1D). These results show that both bZIP60 and ABA-activated signaling may play a role in increasing the efficacy of biocontrol in the HS biotype.

The observation that the exogenously applied ABA enhanced the severity of *P. setariae* by as much as application of herbicide on the HS biotype (Fig. 5F,G) strongly supports the results from transcriptomic analysis that ABA has a key role in the synergy between sethoxydim and *P. setariae* for biocontrol of the HS-GFT biotype. Also, transcription of the HS contig 9248 (bZIP60) was increased by ABA treatment to a level similar to that of the herbicide treatment (Fig. 5I). This strongly indicated that bZIP60 was the common factor between ABA and sethoxydim in enhancing GFT biocontrol. In plants, bZIP60 is the substrate for inositol-requiring enzyme 1 (IRE1), a transmembrane protein that acts as a sensor regulating the unfolded protein response (UPR). The IRE1/bZIP60 branch of UPR plays an essential role in viral infection^46^ and in plant defense responses^47,48^. The current study indicated that bZIP60 may have also suppressed plant defense response during infection of GFT by *P. setariae*.

Protein ubiquitination pathways were also increased as part of the synergy between herbicide and *P. setariae* for GFT biocontrol (Supplementary Table S11). Ubiquitination is a post-translational modification of proteins that regulates various cellular processes in eukaryotes^49^. It is also involved in a step-wise cascade of reactions catalyzed by ubiquitin-activating, -conjugating and -ligation enzymes^50^. The transcription levels of some of these enzymes were also increased by the herbicide + *P. setariae* treatment (Supplementary Table S11), which may have indicated a role of protein ubiquitination in the synergistic interaction.

## Concluding remarks

**Figure 6:**
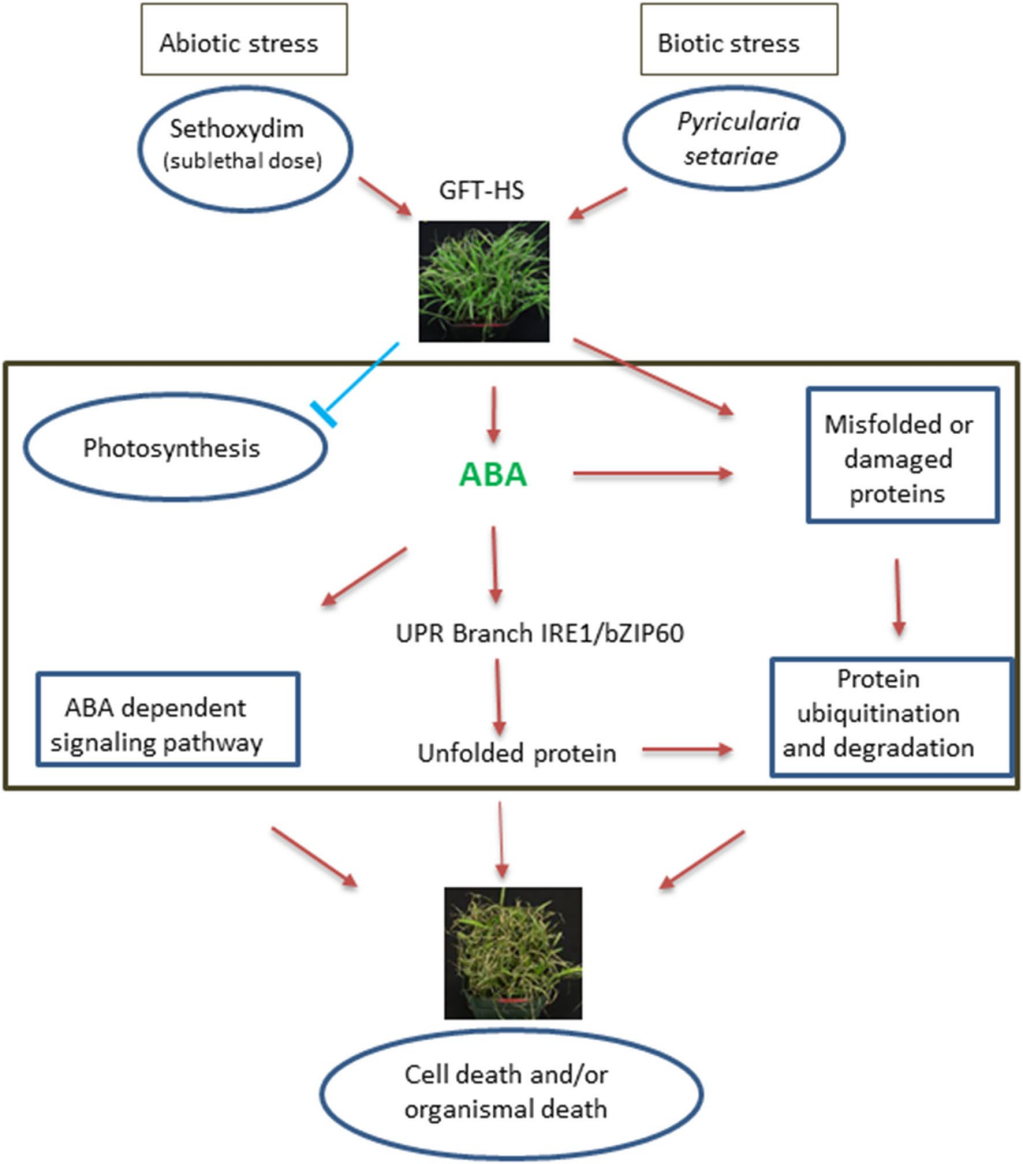
Proposed regulatory model for the herbicide sethoxydim at sublethal dose in synergizing biocontrol of herbicide-sensitive green foxtail by *Pyricularia setariae*.

The current study identified potential mechanisms underlying the synergistic interaction between the herbicide sethoxydim and fungus *P. setariae* in biocontrol of GFT (Fig. 6). Under the stresses imposed by the herbicide at a sublethal dose, photosynthesis was suppressed in HS-GFT plants while ABA-activated signaling and the expression of TF bZIP60 were enhanced. The activation of bZIP60 may play a pivotal role in the synergized biocontrol by acting as a negative regulator of defense response to the infection by *P. setariae*. Even though this study did not identify an improved biocontrol strategy for HR GFT, it demonstrated that RNA-seq can be a useful tool for studying plant-fungus interactions that might lead to improved weed biocontrol.

## Supplementary information

Supplementary Information 1.

Supplementary Information 2.

Supplementary Information 3.

Supplementary Information 4.

Supplementary Information 5.

Supplementary Information 6.

Supplementary Information 7.

Supplementary Information 8.

Supplementary Information 9.

Supplementary Information 10.

Supplementary Information 11.

Supplementary Information 12.

Supplementary Information 13.

## Data Availability

The datasets supporting the results of this article are included in the article, as well as in the supplementary information attached. Sequencing data and de novo assemblies of HS and HR GFT transcriptomes have been placed in NCBI (biosamplehelp@ncbi.nlm.nih.gov) under the BioSample accessions SAMN16338388 and SAMN16338389, respectively. The information is accessible publically.
