# Supplementary material for: Transcriptome analysis identified the mechanism of synergy between sethoxydim herbicide and a mycoherbicide on green foxtail
Source: Sci Rep. 2020 Dec 10;10:21690. doi: 10.1038/s41598-020-78290-6 (PMC7730142; doi:10.1038/s41598-020-78290-6)
Supplement: Supplementary file 1 — Supplementary Information 1. [file 41598_2020_78290_MOESM1_ESM.docx]

**Supplementary Information**

**Fig. S1**. Herbicide sethoxydim at sub-lethal rate synergizes biocontrol of GFT-HS.

**Fig. S2**. Herbicide sethoxydim at sub-lethal rate does not synergize biocontrol of GFT-HR.

**Fig. S3**. Statistics of gene ontology (GO) annotation by Blast2GO. (A) The taxonomic distribution of BlastX top hits for each assembled GFT-HS contig against NCBI nr database. (B) The taxonomic distribution of BlastX top hits for each assembled GFT-HR contig. (C) Pie chart of GFT-HS contigs with different levels of annotation. (D) Pie chart of GFT-HR contigs. (F) The distribution of GO levels annotated ( E) with GFT-HS and (F) with GFT-HR contigs.

**Fig. S4**. Top 20 GO terms annotated with GFT-HS and GFT-HR contigs.

**Fig. S5**. Principal component analysis of RNA-seq data in different treatments.

**Fig. S6**. Validation of RNA-seq data using quantitative PCR (qPCR). The qPCR-fold change of each contig was calculated by comparing the relative quantities of respective treatments to mock, while the RNA-seq fold change was calculated by comparing the RPKM value of each treatment to mock.

**Table S1**. Statistics of all assemblies of GFT transcriptome

**Table S2**. Annotation of HS assembled contigs

**Table S3**. Annotation of HR assembled contigs

**Table S4**. Pairwise tBlastn comparisons of HS and HR assembled GFT transcriptomes with those of four fully sequenced species in Poaceae

**Table S5**. Summary of identified DEGs in HS

**Table S6**. Summary of identified DEGs in HR

**Table S7**. Primers used in quantitative PCR assay

**Table S8**. Biological processes enriched with DEGs

**Table S9**. DEGs involving in ABA synthesis under treatment of synergy

**Table S10**. DEGs of bZIP transcription factors under treatment of synergy

**Table S11**. DEGs involving in protein ubiquitination under treatment of synergy
