# Supplementary figures and images for: Transcriptome analysis identified the mechanism of synergy between sethoxydim herbicide and a mycoherbicide on green foxtail
Source: Sci Rep. 2020 Dec 10;10:21690. doi: 10.1038/s41598-020-78290-6 (PMC7730142; doi:10.1038/s41598-020-78290-6)

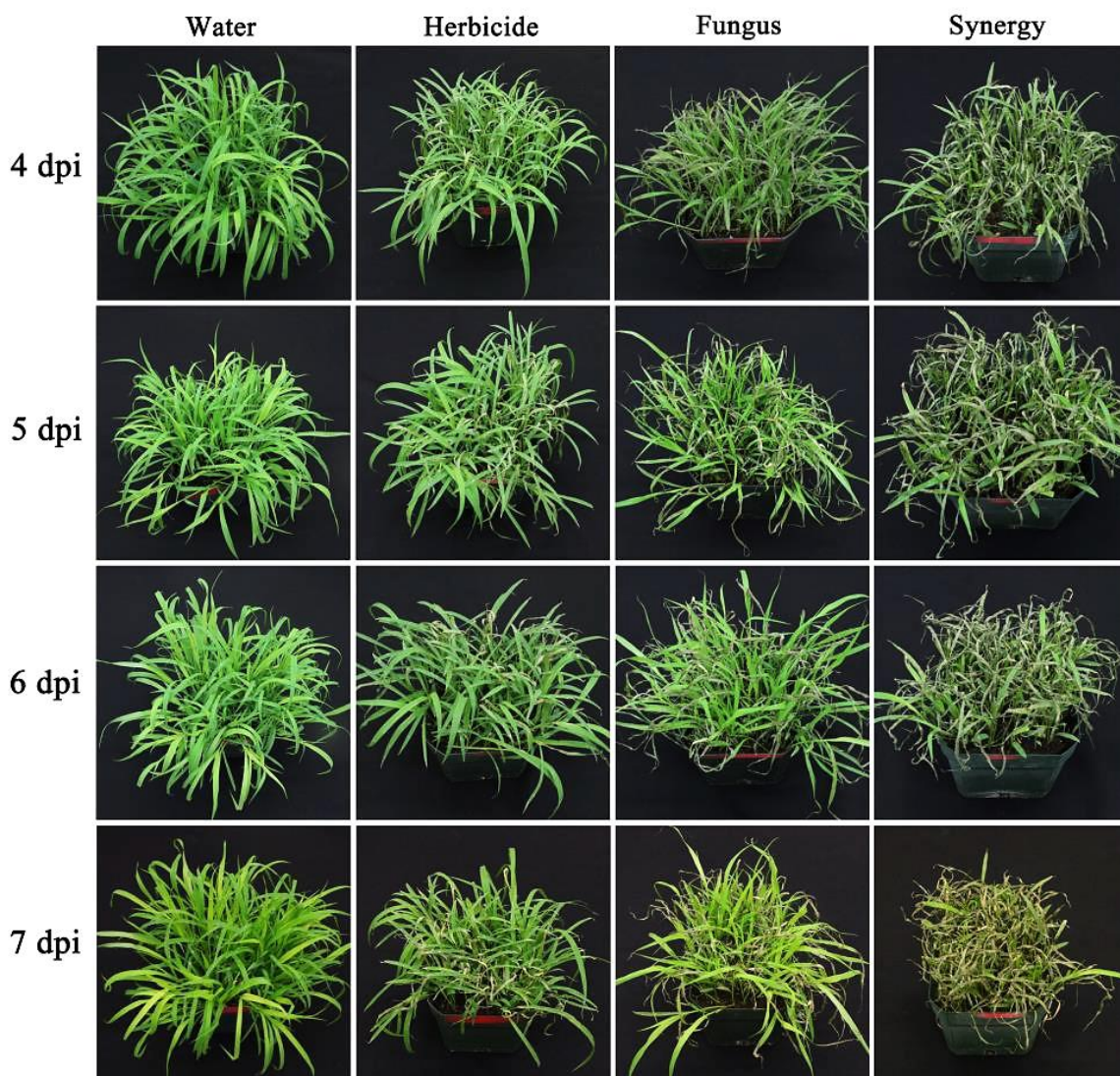

**Figure S1**

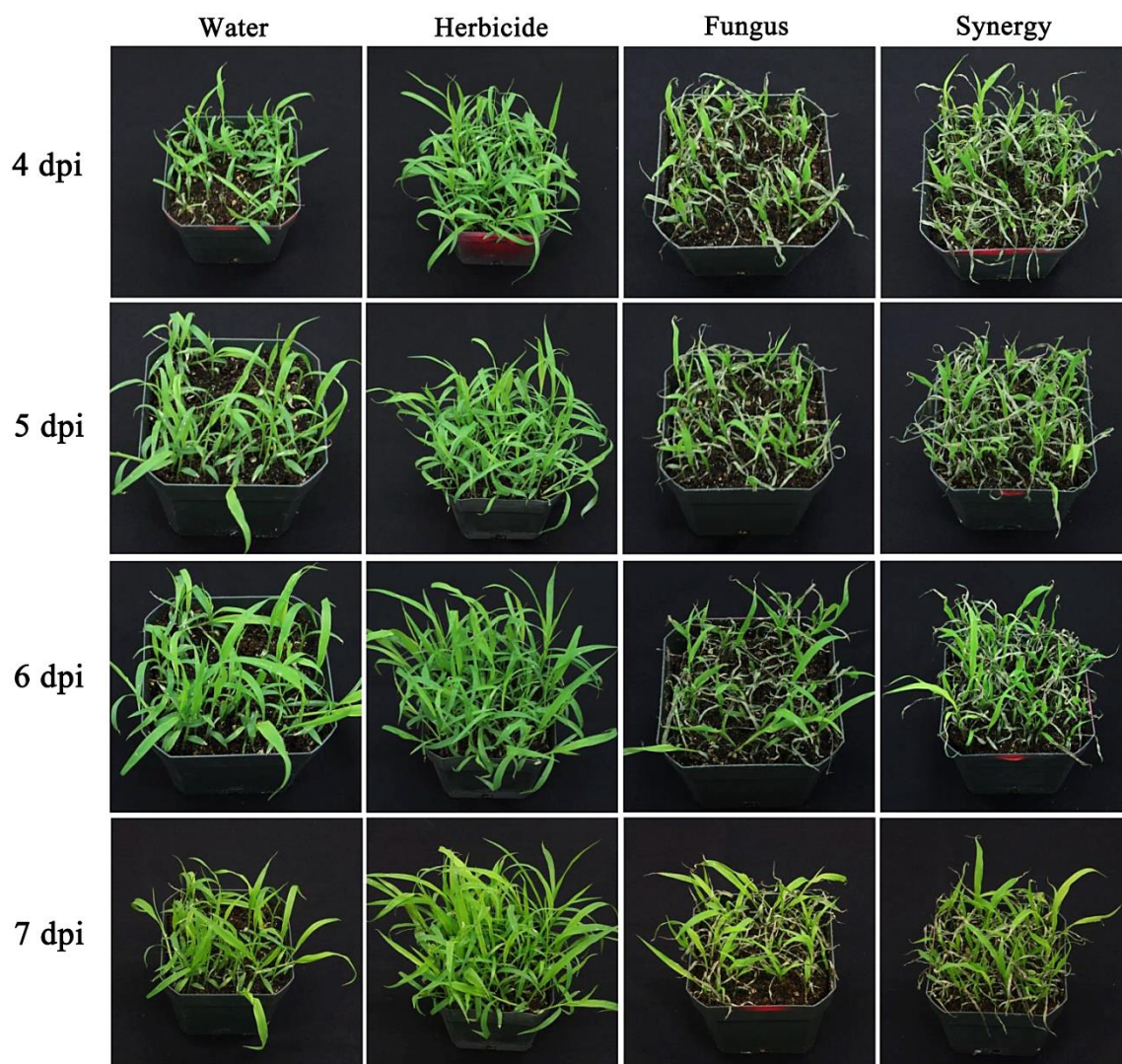

**Figure S2**

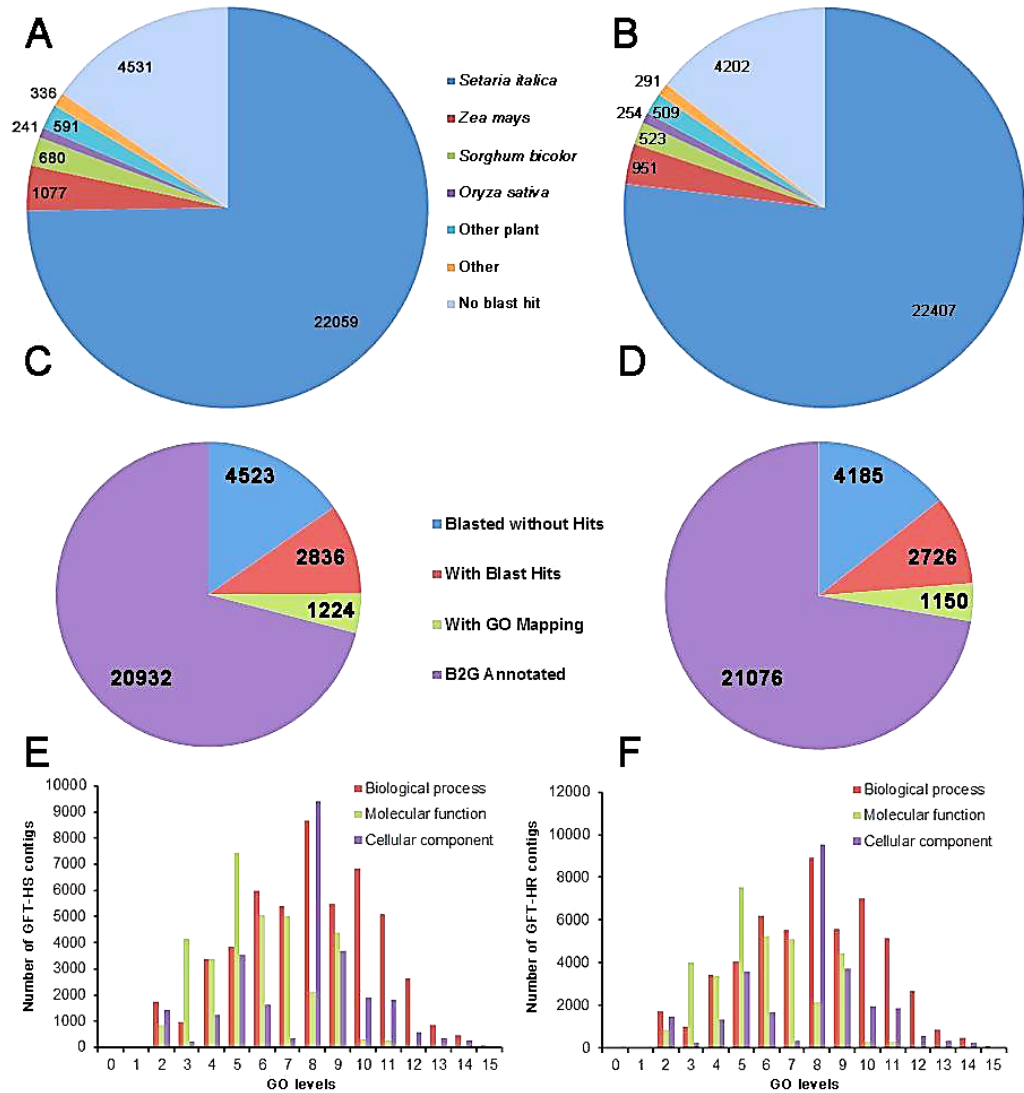

**Figure S3**

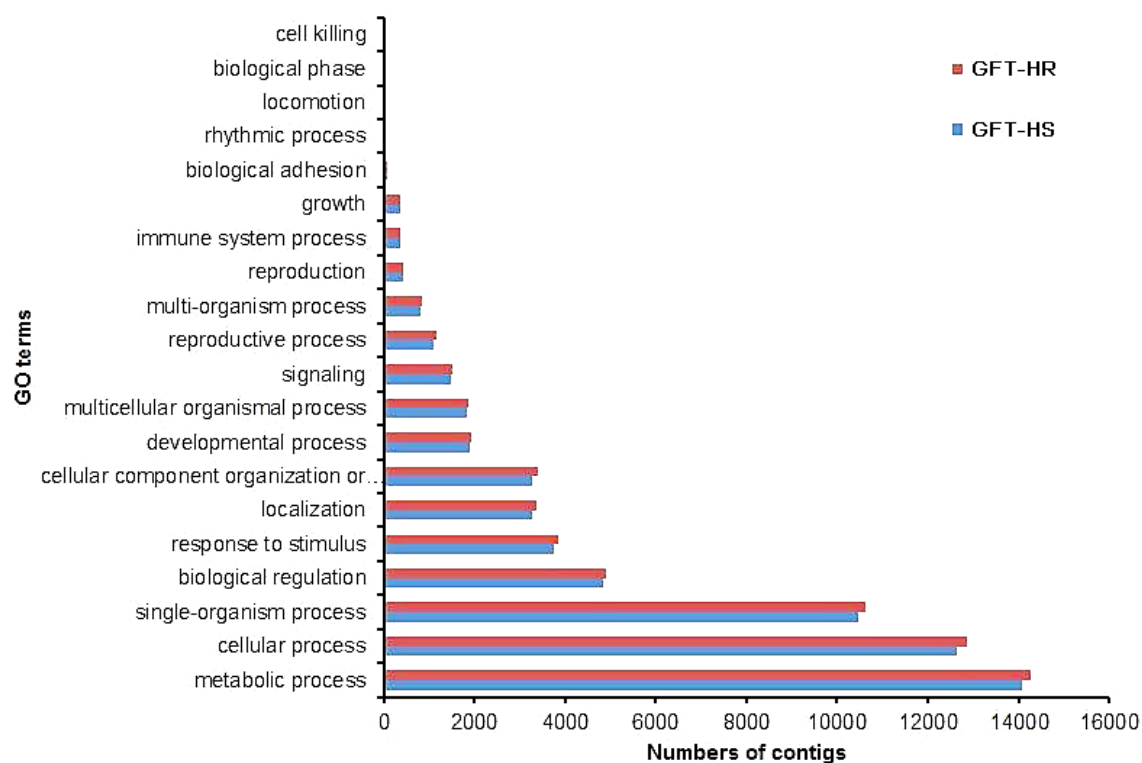

**Figure S4**

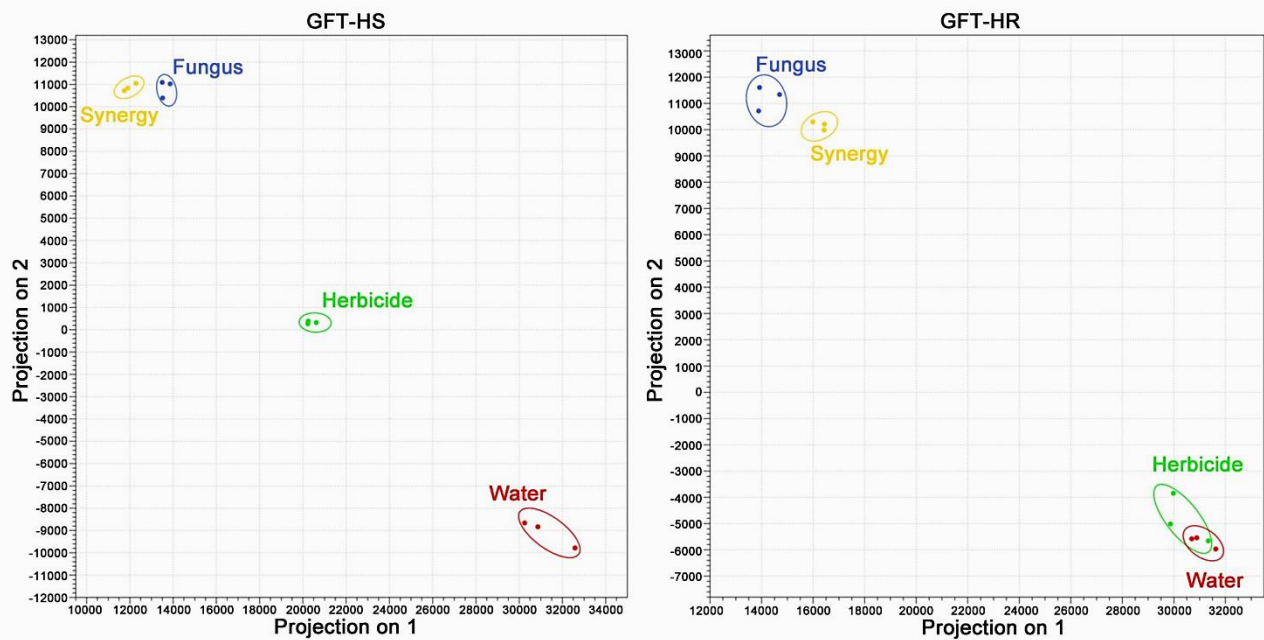

Figure S5

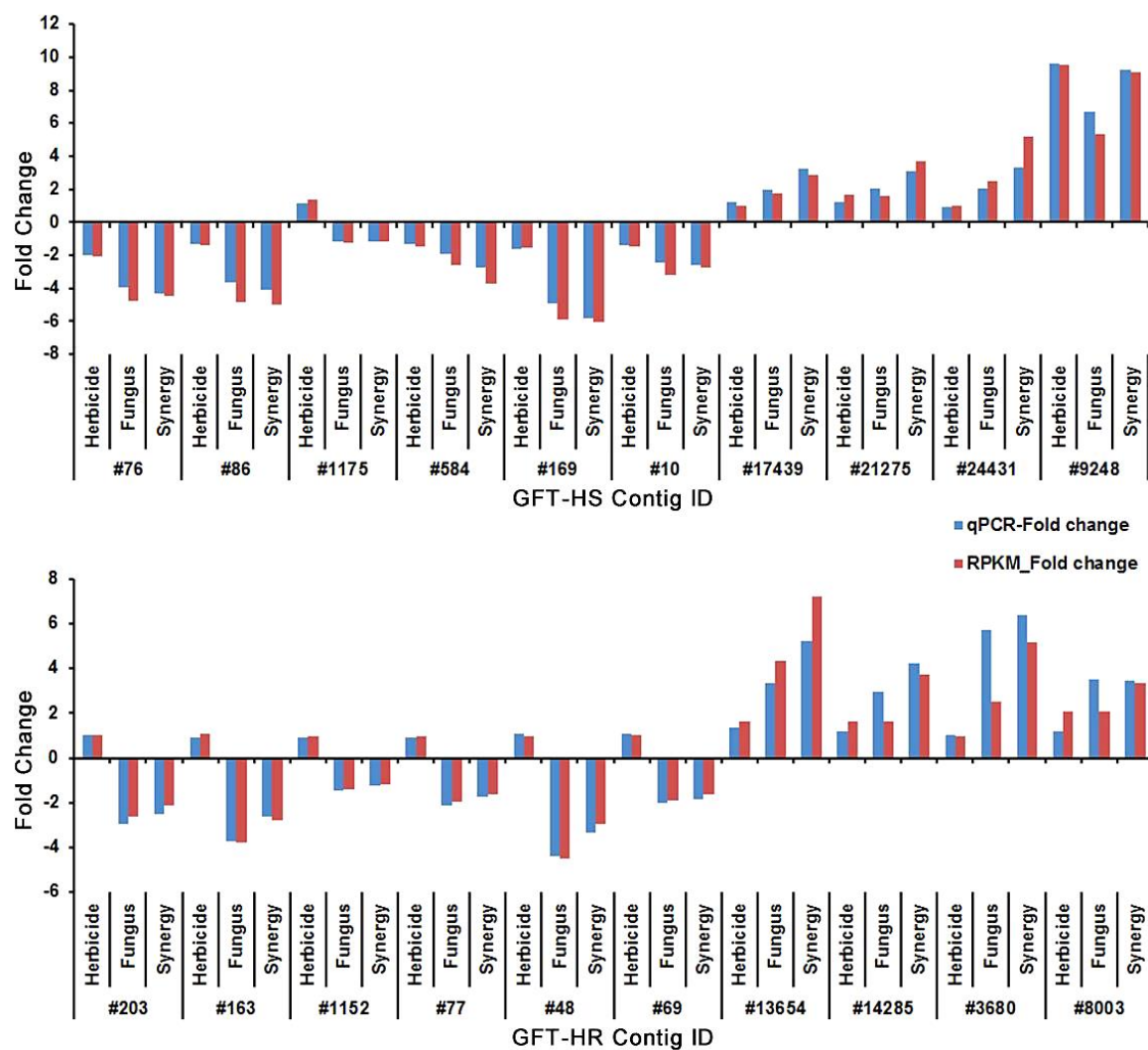

**Figure S6**

Supplement: Supplementary file 2 — Supplementary Information 2. [file 41598_2020_78290_MOESM2_ESM.pdf]
